# Supplementary material for: Generation of iPSC-Derived Human Peripheral Sensory Neurons Releasing Substance P Elicited by TRPV1 Agonists
Source: Front Mol Neurosci. 2018 Aug 22;11:277. doi: 10.3389/fnmol.2018.00277 (PMC6113370; doi:10.3389/fnmol.2018.00277)
Supplement: Supplementary file 12 [file Data_Sheet_3.DOCX]

Supplementary Material

GENERATION OF iPSC-DERIVED HUMAN PERIPHERAL SENSORY NEURONS RELEASING SUBSTANCE P ELICITED BY TRPV1 AGONISTS

**Marília Zaluar P. Guimarães, Rodrigo De Vecchi, Gabriela Vitória, Jaroslaw K. Sochacki, Bruna S. Paulsen, Igor Lima, Felipe Rodrigues da Silva, Rodrigo F. Madeiro da Costa, Newton G. de Castro^2^, Lionel Breton, Stevens K. Rehen^*^**

*** Correspondence:** Corresponding Author: [srehen@lance-ufrj.org](mailto:srehen@lance-ufrj.org)

# Supplementary Data

*1.1-* ***Excel spreadsheet attached* – DATASHEET 1: Raw data immuno quantifications**

# Raw data table of immunostaining quantification: All tables contain important information regarding the assay design such as plate location of each condition (Row and Column), time point, Cell type, Marker, Plate number and Number of analyzed fields. It includes all results from each experiment listed under Total number of cells and Selected nuclei that includes only nuclei not touching the field border, Number of positive cells for specific markers, Average Neurite Length and Fluorescence Intensity. Each result is presented as a group of two in dependent experiments under the label “Plate”.

**1.2 - *Excel spreadsheet attached* - DATASHEET 2: RNA-Seq global expression data in transcripts per million (TPM).**

The RNA-Seq results spreadsheet contains the Ensembl and Gene ID in the first and second columns, respectively, and the normalized gene expression data in transcripts per million (TPM) of 3 replicates per condition. Columns identified by Sample_C1, Sample_C2 and Sample_C3 contain the basal controls PSN expression data. Columns identified by Sample_CM1, Sample_CM2 and Sample_CM3 contain the RNA-Seq results for the PSN cultures treated with human epidermal keratinocytes conditioned media (HEK-CM) during 10 days after NCPC to PSN differentiation.

# Supplementary Figures and Tables

## Supplementary Figures

**Supplementary Figure 1:** Immunofluorescence staining confirming the lack of expression of neuronal differentiation markers in the hiPSC. (A) Peripherin, (B) Islet1, (C) BRN3A and (D) TRPV1 in GM23279A hiPSC cell line. Calibration bar = 100 µm, n=2-3 independent experiments.

**Supplementary Figure 2:** Negative controls for immunocytochemistry of NCPCs with specific markers, obtained by withholding of the primary antibody. Cells were counter-stained with DAPI for nuclei visualization. (A) TRPV1. (B) Peripherin. (C) BRN3A. (D) Pax6. (E) Islet1. (F) Nestin. Calibration bar = 100 µm, n=2-3 independent experiments.

**Supplementary Figure 3:** Negative controls for immunocytochemistry of peripheral sensory neurons with specific markers, obtained by withholding of the primary antibody. Cells were counter-stained with DAPI for nuclei visualization. (A) TuJ1, (B) TRPV1, (C) Peripherin, (D) Islet1. Calibration bar = 100 µm, n=2-3 independent experiments.

**Supplementary Figure 4**: **Characterization of neurons derived from a different hiPSCs line.** In-house derived hiPSCs line was differentiated into NCPCs and consequently differentiated into neurons that expressed both β-TUBULIN III (B) and PERIPHERIN (C). Calibration bar = 100 µm.

**Supplementary Figure 5: Calcium imaging assay in hiPSCs-derived sensory neurons.** Pseudo-color fluorescence image of basal calcium levels (A). Calcium responses in sensory neurons after 100 nM capsaicin (B) and 70 mM KCl treatment (C). Calcium imaging of sensory neurons with conversion of Fura-2 excitation ratio (340/380 nm) to pseudo virtual color, according to the scale on the left of each image. Arrows indicate active cells.

**Supplementary Figure 6**: Principal component analysis of the six samples. A clear separation in expression profile between the two conditions: C (control medium) and CM (conditioned medium) showing a clear separation between the two conditions. Treatment with HEK-CM is a primary contributor to variance, and independent differentiation of cultures was a secondary component of variance. Variance between replicates was higher among HEK-CM treated cultures (CM) compared to PSN (C) cultures.

**Supplementary Figure 7**: MA-plot of PSN gene expression changes induced by HEK-CM treatment. The log2 fold change for HEK-CM treatment was plotted on the y-axis and the average of the counts normalized by size factor is shown on the axis. Each gene is represented with a dot. Genes with an adjusted p value below the 0.1 threshold are shown in red. Most of the genes expressed by PSN were downregulated after the treatment with HEK-CM

**Supplementary Figure 8:** Neuronal markers expressed by hiPSC-derived PSN compared to neuronal markers reported in the literature.

**Supplementary Figure 9**: **Functional protein association network of hiPSC-derived PSN expressed genes with TRPV1.** Hierarchical clustering network of related genes to TRPV1 modulation shows the relationship of TRPV1 with P2RX3, CB1 receptor, CALM 1, 2 and 3. Network nodes represent proteins. Edges represent protein-protein associations. Green lines represent activation; red lines: inhibition; blue lines: binding; pink lines: posttranslational modification; black lines: reaction; yellow lines: transcriptional regulation. Positive effects (arrowheads) and negative effects (block arrows); unspecific effect (ball-head). Colored nodes: query proteins and first shell of interactors.

## 2.2 - Supplementary Table

**Table 1:** Sequencing details of RNA-Seq data-sets. Library preparation was performed for each sample to make four cDNA libraries. Each library was then sequenced with HiSeq using paired-end reads.

| Sample | Organism | Read length [nt] | Seq method | Reads | % of >= Q30 Bases (PF) |
| --- | --- | --- | --- | --- | --- |
| C1* | Human | 101 | paired-end | 62,783,480 | 89.31 |
| C2* | Human | 101 | paired-end | 65,615,154 | 88.94 |
| C3* | Human | 101 | paired-end | 62,914,180 | 88.53 |
| CM1** | Human | 101 | paired-end | 141,866,224 | 88.77 |
| CM2** | Human | 101 | paired-end | 136,813,864 | 88.66 |
| CM3** | Human | 101 | paired-end | 64,765,448 | 88.91 |

*hiPSC-derived Peripheral Sensory Neurons (IPSDSNs). **IPSDSNs treated with HEK-conditioned media 10DIV.
